# Tumor sialylation impedes T cell mediated anti-tumor responses while promoting tumor associated-regulatory T cells

**DOI:** 10.18632/oncotarget.6822

**Published:** 2016-01-05

**Authors:** Maurizio Perdicchio, Lenneke A. M. Cornelissen, Ingeborg Streng-Ouwehand, Steef Engels, Marleen I. Verstege, Louis Boon, Dirk Geerts, Yvette van Kooyk, Wendy W. J. Unger

**Affiliations:** ^1^ Department of Molecular Cell Biology and Immunology, VU University Medical Center, Amsterdam, The Netherlands; ^2^ EPIRUS Biopharmaceuticals, Leiden, The Netherlands; ^3^ Department of Pediatric Oncology/Hematology, Erasmus University Medical Center, Rotterdam, The Netherlands; ^4^ Clinical Research Division, Fred Hutchinson Cancer Research Center, Seattle, WA, USA; ^5^ Department of Pediatrics, Division of Infectious Diseases, ErasmusMC-Sophia Children's Hospital, Rotterdam, The Netherlands

**Keywords:** sialic acid, tumor, immune regulation, regulatory T cells, natural killer cells

## Abstract

The increased presence of sialylated glycans on the tumor surface has been linked to poor prognosis, yet the effects on tumor-specific T cell immunity are hardly studied. We here show that hypersialylation of B16 melanoma substantially influences tumor growth by preventing the formation of effector T cells and facilitating the presence of high regulatory T cell (Treg) frequencies. Knock-down of the sialic acid transporter created “sialic acid low” tumors, that grew slower *in-vivo* than hypersialylated tumors, altered the Treg/Teffector balance, favoring immunological tumor control. The enhanced effector T cell response in developing “sialic acid low” tumors was preceded by and dependent on an increased influx and activity of Natural Killer (NK) cells. Thus, tumor hypersialylation orchestrates immune escape at the level of NK and Teff/Treg balance within the tumor microenvironment, herewith dampening tumor-specific T cell control. Reducing sialylation provides a therapeutic option to render tumors permissive to immune attack.

## INTRODUCTION

Innate immune cells play a crucial role in the immune response against tumors. Particularly, dendritic cells (DCs), macrophages and NK cells are involved in the first phase of the process known as cancer immunoediting, where these cells detect the presence of a developing tumor and coordinate as well as co-operate with the adaptive immune system to eradicate the tumor [1]. Specific activation of naive tumor-specific T lymphocytes by DCs is required to generate an army of effector CD8^+^ and CD4^+^ T cells that can effectively eliminate tumor cells. Activated CD8^+^ T cells acquire effector functions such as cytolytic activity and/or production of interferon (IFN)-γ and tumor necrosis factor (TNF) [2]. Tumor-specific CD4^+^ effector T cells contribute to tumor eradication either via direct tumor cell killing [3] or via the activation of macrophages at tumor sites [4]. Moreover, activated CD4^+^ T cells aid induction of memory CD8^+^ T cells, which can maintain long-term tumor control.

Next to cytotoxic T cells, also NK cells have the capacity to lyse tumor cells. Yet, in contrast to T cells, NK cells represent the first line of defense against transformed cells. Low or absent expression of MHC-I triggers NK cell effector functions, which include release of IFN-γ and cytotoxic granules and induction of apoptosis of target cells. High frequencies of intra-tumoral NK cells have been associated with good prognosis of patients [5]. Hence, alterations in frequency and function of innate and T cells aid tumor immune escape. Indeed, DCs and macrophages in the tumor microenvironment are characterized by an anti-inflammatory or tolerogenic phenotype promoting the induction of anergic and/or regulatory T cells (Treg) [6]. Furthermore, high numbers of both natural as well as induced regulatory T cells (nTregs and iTregs) infiltrate into the tumor site [7, 8] and together with tolerogenic DCs and macrophages they act in concert to hamper the influx and function of effector T cells [7–9].

Tumor cells have been shown to display aberrant glycosylation [10, 11]. In particular, increased levels of sialic acids, driven by enhanced expression and activity of beta-galactoside α2,6-sialyltransferase 1 (ST6Gal-1) and/or α-N-acetylgalactosaminide α2,6-sialyltransferase 1 (ST6GalNAc-I), have been detected, which often correlate with tumor invasion and poor prognosis of malignancies [12–14]. Sialic acids are the outermost monosaccharides on glycan chains of glycoproteins and glycolipids, attached to the underlying glycans with α2,3-, α2,6- or α2,8-linkage [13] and as such the recognition elements for selectins and sialic acid-binding Ig-like lectins (siglecs) [13, 15]. The negative charge of highly abundant sialic acids causes dissociation from the primary tumor and spreading of tumor cells. Binding to selectins expressed on endothelial cells allows tumor cells to disseminate [16–18], whereas binding to siglecs allows interaction with the immune system.

We here investigated whether the increased presence of sialylated glycans on the surface of tumors promotes cellular immune evasion by interfering with the T effector/Treg balance regulated by innate immune cells.

## RESULTS

### Reduction of sialic acids on B16 surface antigens empowers anti-tumor immunity

**Figure 1:**
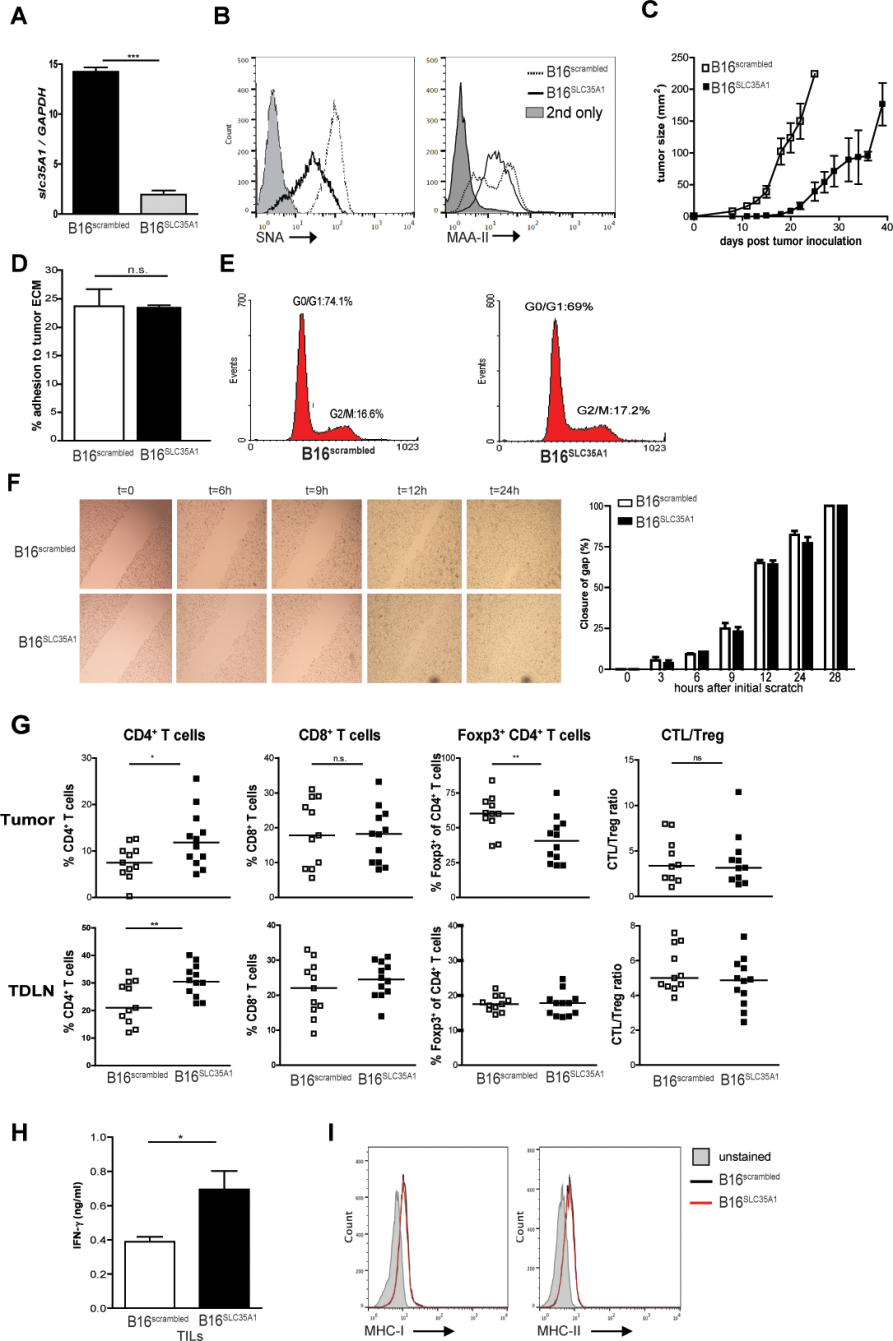
Reduced sialic acid levels on B16 melanoma decline Treg frequencies and increase effector T cell numbers and tumor control (**A**) *slc35A1* gene expression was analyzed in B16^SLC35A1^ and B16^scrambled^ cells by qRT-PCR and normalized to GAPDH; the mean ± s.e.m of duplicate measurements is shown (*n* = 4 independent analyses; ***P < 0.001). (**B**) Detection of a2-6- and a2-3-linked sialic acids using the plant lectins *SNA* and MAA-II on B16^SLC35A1^ (black line) and B16^scrambled^ (dashed line) tumors by flow cytometry. Grey filled histograms represent conjugate control. *n* = 3 independent experiments. (**C**) Tumor growth in B16^SLC35A1^ and B16^scrambled^ tumor-bearing mice (*n* = 7/group), indicated as mm^2^ (mean ± s.e.m). Shown is one of two independent experiments. (**D**) Tumor cell adhesion to matrigel-coated plates. Results shown as percentage of adhering cells (mean ± s.e.m) and represent 3 independent experiments. ns., not significant. (**E**) Cell cycle analysis of B16^SLC35A1^ and B16^scrambled^ tumors by DNA content. Percentage of cells in G0/G1 interphase and G2/M mitotic phase are indicated. Results represent 3 experiments. (**F**) Scratch assay to assess migratory capacity of B16^SLC35A1^ and B16^scrambled^ tumors. Left, Bright-field images (200 ×) of confluent tumor cells showing re-growth following *in-vitro* scratch. Right, quantification of distance between edges of linear scratch. Data represent 3 independent experiments. ns, not significant. (**G**) Percentage of total CD4^+^, CD8^+^ and Foxp3^+^CD4^+^ T-cells as well as CTL/Treg ratio in tumor and TDLN from tumor-bearing mice as detected by flow cytometry at time of sacrifice. Dots represent individual mice (*n* = 11) of 2 independent experiments. Bars indicate median/group, n.s. = not significant; *P < 0.05; **P < 0.01. (**H**) IFN-γ levels secreted by TILs from B16^SLC35A1^ and B16^scrambled^ tumors. Data represent 2 independent experiments, *P < 0.05. (**I**) MHC-I and MHC-II expression on B16^SLC35A1^ and B16^scrambled^ tumors before injection into mice. Plots represent two independent measurements.

To investigate the influence of tumor-expressed sialic acids on anti-tumor immunity we reduced their presence on the hyper-sialylated cell surface of murine B16 melanoma by knockdown of the *Slc35a1* gene encoding the Golgi-based CMP-sialic acid transporter [19]. Using this strategy, B16 cells with sustained reduction of surface sialylation were created, which in contrast to other methods such as sialidase treatment, permits long-term immune monitoring in the tumor. Importantly, the sialic acid content of the B16 remains unaffected, only the addition of the sialic acid residues to growing glycan chains on glycoproteins and glycolipids is impeded. *Slc35a1* knockdown was confirmed using qRT-PCR and specifically decreased the quantity of α2,6-linked sialic acids on the B16 surface compared to B16 cells treated with a non-targeting shRNA (hereafter called B16^SLC35A1^ and B16^scrambled^, respectively) as shown using the plant lectins *Sambucus Nigra* and *Maackia Amurensis* (SNA and MAA-II, Figure 1A, 1B). B16^scrambled^ tumors were already visible on day seven after injection into immunocompetent C57BL/6 mice and grew substantially faster and larger than sialic acid low B16^SLC35A1^ tumors, which became detectable around day 15 and remained much smaller in size for a prolonged period (Figure 1C). Since aberrant sialylation has been correlated with the invasive properties of tumors, we evaluated whether the reduction of α2,6-sialic acids on B16 altered these characteristics *in-vitro*. However, no difference between B16^SLC35A1^ and B16^scrambled^ tumor cells in binding to plates coated with tumor-derived extra-cellular matrix components could be observed (Figure 1D). Additionally, the proliferative capacity was not affected as indicated by cell-cycle analysis (Figure 1E). Using the scratch assay we ascertained that the B16^SLC35A1^ and B16^scrambled^ tumors migrated at the same rate and closed the gap within 28 hours (Figure 1F). B16^scrambled^ tumors showed comparable characteristics to unmodified WT B16 tumor cells (Supplementary Figure 1A–1D).

As a reduction of α2,6-sialic acids on B16 surfaces did not alter tumor intrinsic characteristics *in-vitro*, we hypothesized that the reduced growth of B16^SLC35A1^ tumors *in-vivo* arose from changes in the host's anti-tumor immune response. At time of sacrifice, significant higher CD4^+^ T cell numbers were detected within the tumor-infiltrating lymphocytes (TILs) and tumor-draining lymph nodes (TDLN) of B16^SLC35A1^ tumors (Figure 1G). Notably, in the B16^SLC35A1^ microenvironment the fraction of Foxp3^+^ within the CD4^+^ T cell population was strongly reduced. Together with the elevated IFN-γ levels secreted by B16^SLC35A1^-infiltrating lymphocytes upon *ex-vivo* re-stimulation (Figure 1H), these findings suggest that the CD4^+^ and CD8^+^ T cells in B16^SLC35A1^ tumors are effector rather than tolerogenic T cells. Despite the strong reduction in Foxp3^+^ T cells within the CD4^+^ T cell population at this stage of tumor growth, the CTL/Treg ratio in the B16^SLC35A1^ tumor was not different from that in B16^scrambled^ tumors (Figure 1G). In contrast to our observation on TILs, no reduction in Foxp3^+^CD4^+^ T cells was found in TDLNs of B16^SLC35A1^ tumors (Figure 1G). Furthermore, no significant differences in T cell numbers and phenotype were observed in the spleens of tumor-bearing mice, indicating that anti-tumor immunity is confined to the tumor-microenvironment (Supplementary Figure 1E). Furthermore the comparable low MHC-I and MHC-II expression levels on the two tumor cell lines rules out improved recognition of B16^SLC35A1^ by tumor-reactive CD8^+^ and CD4^+^ T cells (Figure 1I).

Together, these findings clearly show that a reduction of sialic acids on tumor surfaces evokes a switch in the type of immunity in the tumor area: instead of immunosuppressive Treg IFN-γ-producing TILs cells are present that may control tumor growth.

### Sialic acids on tumors dampen activity of NK cells

**Figure 2:**
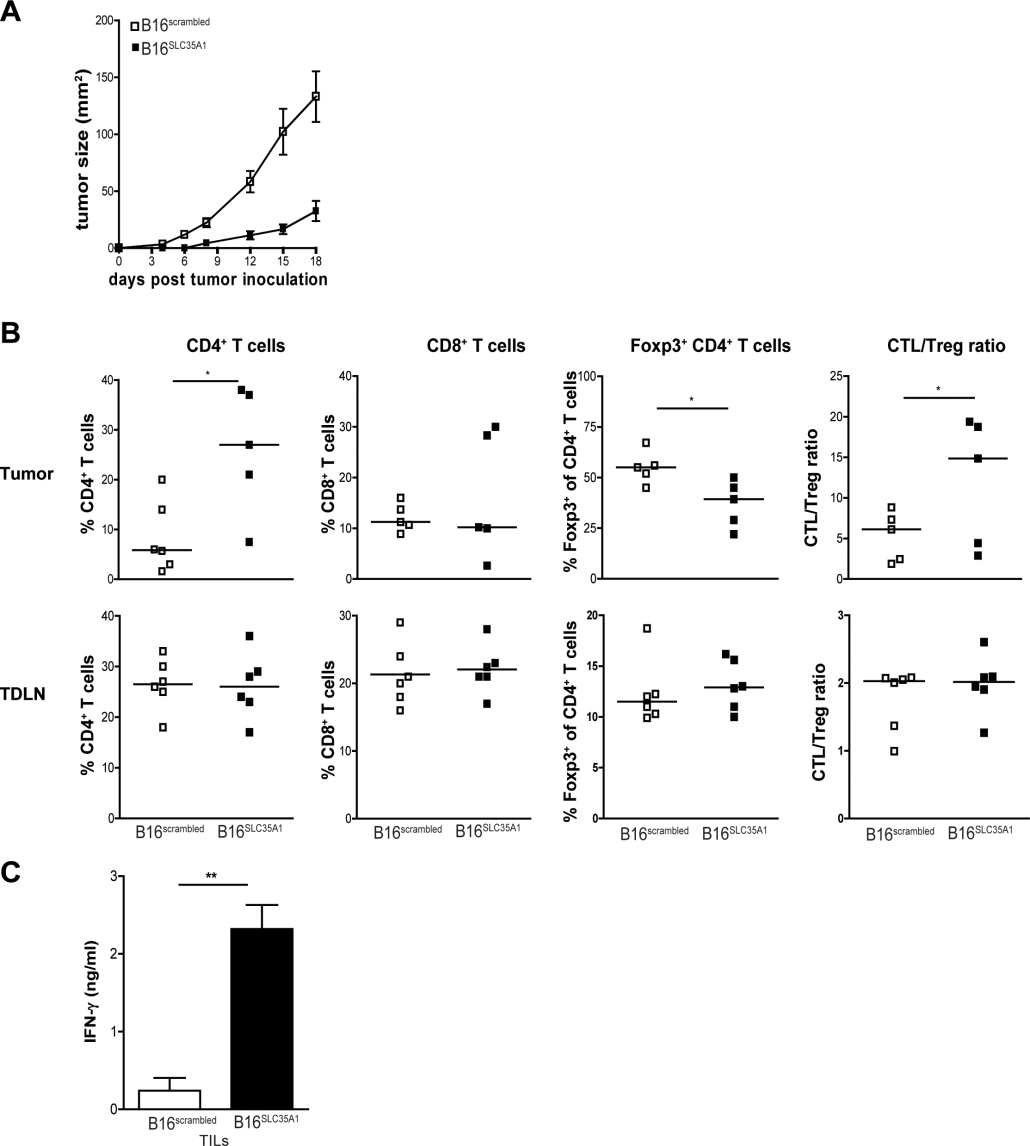
*SLC35A1* knockdown in tumors alters intra-tumoral CD4^+^ Tregs frequencies at early stages of tumor development Mice (*n* = 6/group) were inoculated with either B16^SLC35A1^ and B16^scrambled^ tumors and (**A**) tumor growth represented in mm^2^ (mean ± s.e.m) was measured every 2–3 days until day 18. (**B**) Mice were sacrificed 18 days after tumor injection and percentages of total CD4^+^ T cells, CD8^+^ T cells, and of Foxp3^+^CD4^+^ T cells in the tumor and TDLNs from tumor-bearing mice as detected by flow cytometry. CTL/Treg ratios were calculated for each mouse. Dots represent individual mice (*n* = 6 mice/group; n.s. = not significant; *P < 0.05). Bars indicate median of each group. Statistical significance is indicated (Student's *t* test). (**C**) Levels of IFN-γ secreted by TILs from B16^SLC35A1^ and B16^scrambled^ tumors upon PMA/ionomycin activation (mean ± s.e.m.; **, *P* < 0.01). Data are representative of 2 independent experiments.

To analyze whether the delayed growth of B16^SLC35A1^ tumors *in-vivo* was paralleled by variations in innate cell subsets, which are predominantly involved in the first phases of cancer immunoediting, mice were sacrificed 18 days after tumor implantation, coinciding with early phases of B16^SLC35A1^ tumor development (Figure 2A). Analysis of the T cells within B16^SLC35A1^ tumors revealed the same increase in total CD4^+^ T cells found at a later stage (*i.e.* 40 days after implantation; Figures 2B and 1G) as well as a markedly lower Foxp3^+^ fraction within the CD4^+^ T cell compartment than in B16^scrambled^ tumors (Figure 2B). No significant changes in tumor-infiltrating CD8^+^ T cells were detected at this early stage of tumor development (Figure 2B). Similar as before the reduced frequency of CD4^+^ Tregs in B16^SLC35A1^ tumors was concomitant with increased IFN-γ production by infiltrating lymphocytes, indicating a shift from T cell tolerance towards T cell immunity that sets stage early after tumor implantation (Figure 2C). This is also underlined by the significant higher CTL/Treg ratio in the B16^SLC35A1^ tumors compared to that in B16^scrambled^ tumors at this early stage of B16^SLC35A1^ tumor growth (Figure 2B).

**Figure 3:**
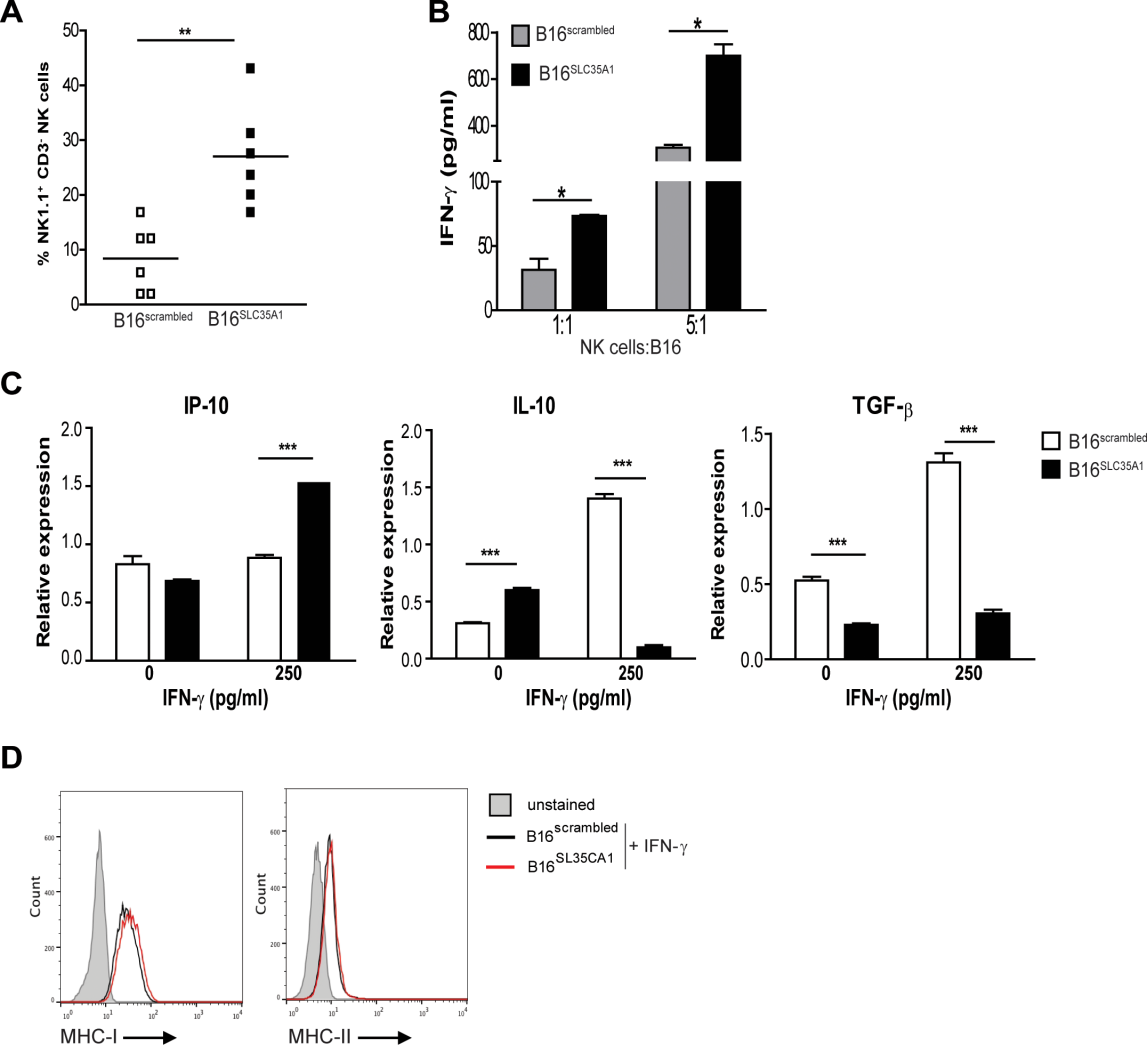
Increased numbers of intra-tumoral NK cells ignite an immunogenic milieu in B16SLC35A1 tumors via IFN-γ (**A**) Percentage of NK cells, distinguished as NK1.1+CD3- cells, within B16^SLC35A1^ and B16^scrambled^ tumors (*n* = 6 mice/group) using flow cytometry. Bars indicate the median of each group; **P < 0.05. (**B**) NK cells, enriched from naive splenocytes, were incubated at indicated E:T ratio's from B16^SLC35A1^ and B16^scrambled^ tumors and with IFN-γ was determined by ELISA from the supernatant 4 h later. Data are representative of 3 experiments; *P < 0.05. (**C**) IP-10, IL-10 and TGF-β mRNA levels expressed by B16^SLC35A1^ and B16^scrambled^ tumors in the presence or absence of rm-IFN-γ (*n* = 4 independent experiments; ***P < 0.001). (**D**) Flow cytometric analysis of MHC-I and MHC-II expression on B16^SLC35A1^ and B16^scrambled^ cell surface 24 h after culture in the presence of 250 pg/ml rm-IFN-γ. Plots represent two independent measurements.

Interestingly, B16^SLC35A1^ tumors contained 25% of NK1.1^+^CD3^−^ NK cells, while in B16^scrambled^ tumors only 10% was detected (Figure 3A). NK cells are amongst the first responders during tumor development, where they actively kill the transformed cells by secreting IFN-γ and/or release of cytotoxic granules. This function may be inhibited in hypersialylated tumors as indicated by recent *in-vitro* studies [24, 25]. Indeed, the IFN-γ levels in the supernatants of *in-vitro* NK and tumor cell co-cultures clearly demonstrate that a reduction in sialic acids increased the IFN-γ production by NK cells, at each E:T ratio tested (Figure 3B). In addition to its cytolytic effects, IFN-γ can also induce tumor cells to produce chemokines that attract immune effector cells, such as CXCL10 (IP-10) [20, 21]. The presence of IFN-γ increased the levels of IP-10 mRNA in B16^SLC35A1^ but not in B16^scrambled^ (Figure 3C). Of note, both tumor types expressed equal levels of IFN-γ receptor mRNA (Supplementary Figure 1F). Furthermore, we found that in contrast to B16^scrambled^, B16^SLC35A1^ did not increase IL-10 and TGF-β mRNA levels in the presence of IFN-γ, thus making the tumor micro-milieu of the sialic acid low tumor less tolerogenic (Figure 3C). IFN-γ also resulted in an significantly increased expression of MHC-I on B16 cells, however, no differences between B16^scrambled^ and B16^SLC35A1^ were observed (Figure 3D). Similar observations were made for MHC-II expression levels, although these were only moderately increased by IFN-γ.

Thus, hyper-sialylated tumor cells paralyze the inflammatory program of NK cells and herewith maintain a tolerogenic environment.

### Presence of NK cells is required to evoke the switch in T cell response in sialic acid^low^ tumors

**Figure 4:**
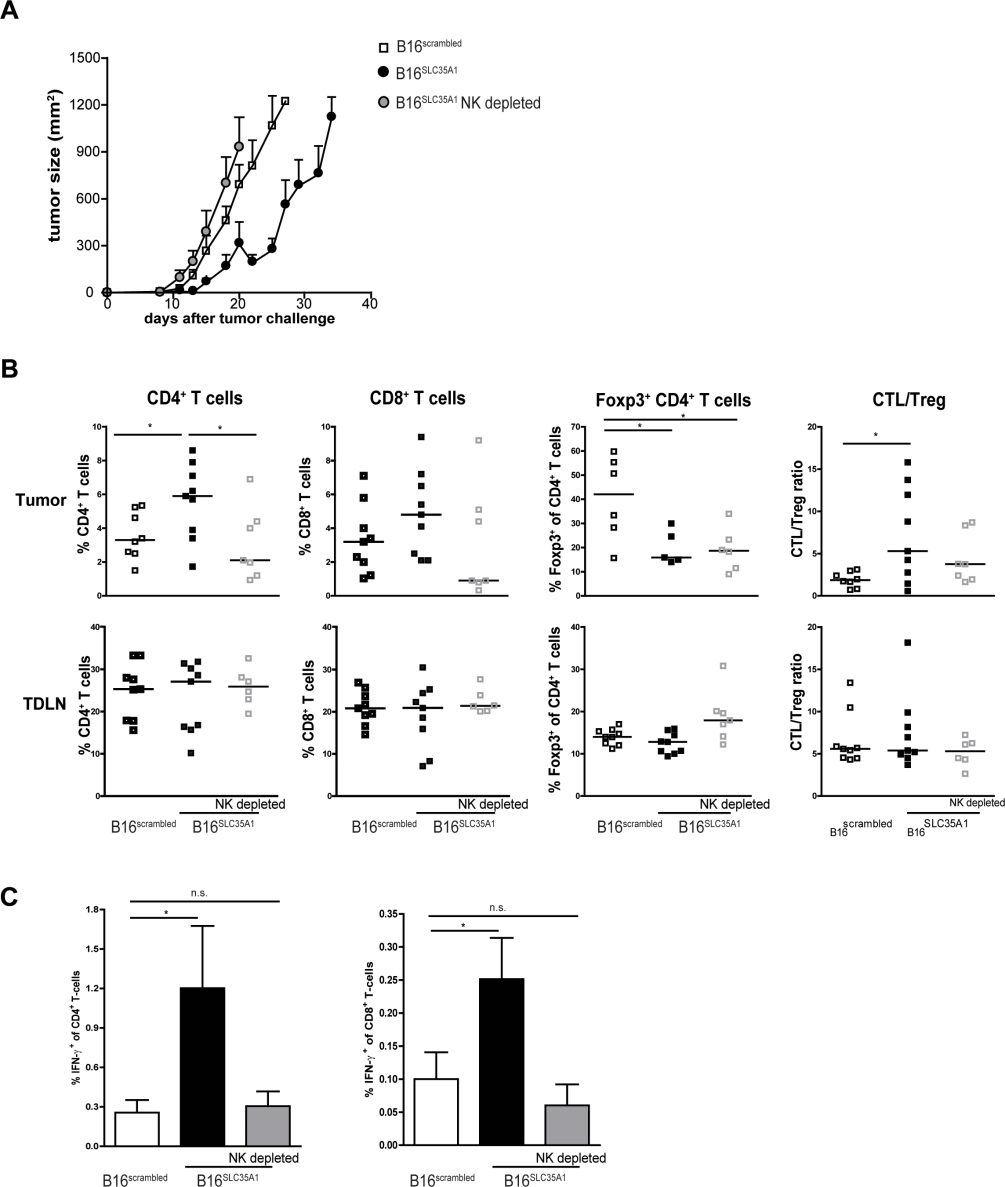
Depletion of NK cells in sialic acid^low^ tumor bearing mice abolishes induction of anti-tumor immunity (**A**) NK-depleted mice were challenged with B16^SLC35A1^ tumor cells; WT mice were challenged with B16^SLC35A1^ or B16^scrambled^ tumor cells. Tumor growth was assessed at different days after tumor inoculation, indicated as mean mm^2^ ± s.e.m; *n* = 7–9 mice/group. Data represent results from two independent experiments. (**B**) TILs and TDLNs were analyzed by flow cytometry to determine the frequency of CD4^+^ and CD8^+^ T cells and of Foxp3^+^ of CD4^+^ T cells. CTL/Treg ratio was calculated for each mouse. Dots represent individual mice. Bars indicate median of each group (*n* = 7–9 mice/group). (**C**) IFN-γ production by activated CD8^+^ and CD4^+^ T cells in TDLN was determined by intracellular staining after PMA/ionomycin restimulation *ex vivo*. Dots represent individual mice; *n* = 5 mice/group. *P < 0.05, **P < 0.01, ***P < 0.001. Graphs shown are representative of two independent experiments.

The presence of higher numbers of IFN-γ-secreting NK cells in B16^SLC35A1^ tumors (Figure 3A) as well as the immunogenic effect of IFN-γ on the tumor milieu, suggests that NK cells play a relevant role in the control of B16^SLC35A1^ growth. To test this, mice challenged with B16^SLC35A1^ tumors were depleted of NK cells by treatment with anti-NK1.1 antibodies (Supplementary Figure 1G). As observed previously, B16^SLC35A1^ tumors grew slower and were smaller in size when implanted in WT NK-competent mice, leading to survival of mice 35 days after tumor transplantation (Figure 4A). However, NK depletion drove B16^SLC35A1^ tumors to grow at a similar rate as B16^scrambled^ tumors (Figure 4A). Of note, growth of B16^scrambled^ tumors was only slightly enhanced when NK cells were depleted (Supplementary Figure 1G). Furthermore, the increased infiltration of CD4^+^ and CD8^+^ T cells into B16^SLC35A1^ tumors was absent when NK cells were depleted (Figure 4B). This was mirrored by the absence of effector T cells in these mice: IFN-γ-producing CD4^+^ and CD8^+^ T cells were only detected in B16^SLC35A1^ tumors (Figure 4C). Although depletion of NK cells did not alter the proportion of Foxp3^+^CD4^+^ T cells within B16^SLC35A1^ tumors, the CTL/Treg ratio was only in favor of tumor control in NK replete B16^SLC35A1^. In contrast to the tumor environment, Treg numbers were significantly higher in the TDLN of NK cell depleted B16^SLC35A1^ tumor bearing mice (Figure 4B).

In summary, these data unveil a clear and versatile role for the levels of sialic acids on tumors regulating NK cell mediated initiation of potent anti-tumor immunity.

## DISCUSSION

Here we show that reducing sialic acids on the surface of melanoma cells evokes the generation of large numbers of effector T cells that infiltrate the tumor area and simultaneously dampens the intra-tumoral presence of Foxp3^+^ Tregs, together curtailing tumor growth. Furthermore, we provide evidence that this switch in T cell response from tolerogenic to immunogenic in sialic acid^low^ melanomas is ignited by NK activation as their depletion abolishes effector T cell induction.

In this study we reduced the amounts of sialic acids on the murine B16 melanoma surface to assess the impact of alterations in sialic acids on the anti-tumor immune response. *Slc35A1* knock down significantly reduced the presence of α2,6-linked sialic acids on the B16 surface. Besides melanoma, also other invasive and metastatic tumors (e.g. colon and breast carcinoma) present an aberrant expression of α2,6-sialic acids mainly because of the overexpression of ST6Gal1 sialyltransferase, which adds terminal sialic acid moieties on N-linked glycans [14]. It has been shown that enhanced expression of α2,6-sialic acid on tumors promotes cell detachment from the tumor mass as well as their invasiveness by facilitating interactions with matrix proteins [22, 23].

We observed that *in-vivo* B16^SLC35A1^ tumors grew significantly slower and remained much smaller in size than B16^scrambled^ tumors for a long period, resulting in better survival of mice bearing B16^SLC35A1^ tumors. Since *in-vitro* intrinsic properties of B16-OVA such as migratory and adhesion abilities were not affected by the reduction of α2,6-sialic acids, the delayed growth of B16^SLC35A1^ tumors *in-vivo* is suggested to be the outcome of improved immune control. This is also underlined by our findings that CD4^+^ T cell numbers were increased in the tumor and TDLNs as well as the ability of both CD4^+^ and CD8^+^ T cells to produce IFN-γ. At the same time, frequencies of tumor-associated Tregs were strongly diminished in the B16^SLC35A1^ tumors. Thus, reducing sialic acids on the tumor surface switches the function of tumor-specific T cells from tolerogenic to effector. Notably, both in the early as well as the later stages of tumor growth, the effects of sialic acid^low^ tumors on T cell frequency and function were mainly evident in the tumor and not in the TDLN, which points to strong intra-tumor immune regulation that is sufficient to control tumor growth.

This study is the first to show the immunological consequences of tumor cell hyper-sialylation. We demonstrated that the presence of NK cells, Tregs, effector T cells, and probably DCs, is affected and that these immune cells cooperate in their mode of action, herewith linking hypersialylation with tumor induced immune tolerance. The sialic acid binding receptors or siglecs are predominantly found on innate immune cells, such as antigen presenting cells (APCs) and NK cells [15]. On APCs cells, siglecs function as endocytic receptors and regulate their activation and cytokine secretion. The CD33/Siglec-3-related siglec (hCD33rSiglecs) subgroup of siglecs possess immunoreceptor tyrosine-based inhibitory motifs (ITIMs) in their intracellular portion, which can counteract the activating signals triggered by receptors containing immunoreceptor tyrosine-based activatory motifs (ITAMs) [15]. This suggests that engagement of hCD33rSiglecs on innate cells by sialylated antigens may result in suppressive effects on the innate immune response. Since on the engineered B16^SLC35A1^ tumors mostly α2,6-linked sialic acids were reduced, together with our observation that the immune response is affected at multiple cellular levels underscores the possibility that different siglec receptors expressed on NK, Tregs and DCs may mediate sialic acid-tumor immune evasion.

Analysis of the immune composition in the early stage of tumor growth revealed increased frequencies of NK cells in B16^SLC35A1^ tumors compared to B16^scrambled^ tumors. Moreover, NK cells engaging B16^SLC35A1^ tumor cells secreted elevated amounts of IFN-γ compared to those engaging B16^scrambled^ cells. A similar enhanced IFN-γ-secretion by NK cells was reported in a study in which sialylation of the chemically induced fibrosarcomas was reduced via sialidase treatment or when the sialylation status of tumor cell surfaces was remodeled using synthetic glycopolymers [24, 25]. Murine NK cells express siglec receptors such as Siglec-E and Siglec-G [26, 27], which bear specificity for α2-6-linked sialylated branches [28]. Thus, it is possible that the reduction of α2,6-sialic acids on B16 tumor cells diminished siglec-mediated inhibition of NK cell functions. Indeed, recent studies showed that *in-vitro* the susceptibility of hypersialyated tumor cells to NK cytotoxicity could be blocked by siglec-blocking antibodies or de-sialylation of the tumors [24, 29]. The absence of this inhibitory interaction paves the way for the NK activating receptor NKG2D with its ligands on tumors, enhancing IFN-γ secretion and killing capacity of NK cells [25]. The augmented numbers of NK1.1^+^ NK cells present in sialic acid^low^ tumors during the early stages of tumor development could be a result of initial activated NK cells producing IFN-γ since IFN-γ has been shown to mediate recruitment of NK cells to sites of infection [30]. This NK cell influx is likely further enhanced by the chemokine IP-10, which was highly induced by IFN-γ in sialic acid^low^ tumors. Furthermore, the increased numbers of NK cells could also be due to reduced frequency of Tregs in the tumor area, as it has been shown that Tregs limit expansion of NK cells by restraining IL-2 from effector T cells [31].

The absence of CD4^+^ and CD8^+^ effector T cells in the B16^SLC35A1^ tumor microenvironment when NK cells were depleted elucidates that the IFN-γ released by activated NK cells likely also functions to activate DCs [32], which obviously are instrumental in the subsequent induction of large amounts of effector T cells. Additionally, the released IFN-γ makes the tumor micro-milieu susceptible for immune attack as we showed that IFN-γ reduced production of anti-inflammatory cytokines by tumor cells. Next to the IFN-γ released by activated NK cells, DC activation may also be the result of diminished siglec triggering on DCs. Similar to NK cells, DCs also express siglecs, and predominantly Siglec-E. We recently showed that Siglec-E-mediated internalization of sialylated antigens by DCs provides them with a tolerogenic phenotype: these DCs promote the induction of Treg. Additionally, the production of pro-inflammatory cytokines and ability to induce effector T cell generation is significantly reduced in these DCs (Perdicchio et al., manuscript under submission). Furthermore, by secreting high levels of sialylated gangliosides tumor cells inhibit the expression of costimulatory molecules on DCs and herewith reduce T cell proliferation [33]. Also, binding of sialylated pathogens to siglecs on DCs has been described to dampen secretion of pro-inflammatory cytokines [34]. DC binding of sialic acid structures on *C. jejuni* modulates the signals DCs provide to naive T cells and that mediate T cell activation and polarization. Thus, it is likely that enhanced tumor sialylation adds to the generation of tolerogenic tumor-associated DCs, which in the tumor microenvironment convert naive T cells into Tregs. This is also suggested by the finding that sialic acid^low^ tumors were inhabited by lower amounts of Tregs than hyper-sialylated tumors. Unexpectedly, in sialic acid^low^ tumors that are depleted of NK cells the frequency of Foxp3^+^ Tregs did not return to the levels observed in sialic acid^high^ tumors. We hypothesized that Tregs expressing NK1.1 could potentially be co-depleted by the anti-NK1.1 Ab treatment. However, this appeared not to be the case as we found that the Foxp3^+^ T cells present in the tumors and TDLN lack expression of NK1.1 (Supplementary Figure 1I). Moreover, Treg frequencies were increased in the TDLN of NK cell depleted mice bearing sialic acid^low^ tumors (Figure 4B). A decreased amount of Tregs in sialic acid^low^ tumors may alternatively result from impaired local expansion of natural Tregs or impaired recruitment due to reduced expression of Treg-attracting chemokines [8, 35]. Which of these scenarios is involved in creating the tolerogenic tumor milieu is subject of current research.

Our data strongly indicate that reducing sialylation may provide a therapeutic option to render tumors permissive to immune attack. This could for example be accomplished by targeted delivery of an sialic acid-blocking glyco-mimetic, which was recently shown to delay metastasis of B16 melanoma when used as adjuvant therapy [36]. Additionally, the use of synthetic small molecules to inhibit sialyltransferase or sialyltransporter activity would also allow to tackle tumors that express α2,3- or α2,8-linked sialic acids or sialyl-Le^X^ [13]. However, whether α2,3- or α2,8-linked sialic acids or sialyl-Le^X^ on tumors have similar effects on the immune composition in the tumor environment remains to be examined.

In conclusion, we showed a correlation between melanoma hyper-sialylation and the induction of a tolerogenic tumor microenvironment marked by high amounts of Tregs and tolerized NK cells. Moreover, our studies unveiled a crucial role for activated NK cells igniting different pathways that result in the generation of tumor-specific effector T-cells. Hence, the reduction of sialic acids on tumors or blocking siglecs on NK cells and/or DCs might have therapeutic implications in immunotherapy of tumors.

## MATERIALS AND METHODS

### Cells and lentiviral transduction

B16-OVA (murine melanoma; kindly provided by Dr. T. Schumacher, National Cancer Institute, Amsterdam, The Netherlands) were cultured in DMEM supplemented with 10% FCS, 50 U/ml penicillin and 50 μg/ml streptomycin (BioWhittaker, Walkersville, MD).

Sialic acid low B16-OVA (hereafter named B16^SLC35A1^) were generated by transducing B16 with the pLKO.1 lentiviral vector containing SLC35A1shRNA. As a control, B16-OVA cells were transduced with pLKO.1 containing non-target shRNA (designated B16^scrambled^). Lentiviral vectors were produced as described earlier [37]. In short, HEK293T cells were co-transfected with pLKO.1-shRNA and the packaging plasmids (pVSV-G, pMDL and pRev-RRE) in the presence of calcium phosphate. One day later, medium was replaced by serum-free medium and culture supernatant was harvested another day later. B16-OVA were seeded at 10^5^ cells/well and transduced with a fixed amount of lentiviral vector when 80% confluent. The day following infection, target cells were selected with 1 μg/ml puromycin.

### Tumor experiments

For *in vivo* studies, 1 × 10^5^ tumor cells were inoculated subcutaneously into the flank of C57BL/6 mice (8-12 weeks old; Charles River Laboratories). Mice were sacrificed when tumors reached a diameter of 1.5 cm and tumors, spleens and lymph nodes were removed for analysis by flow cytometry. To deplete NK cells, mice were injected i.p. with anti-NK1.1 antibodies (PK136; mouse IgG2a; 0.2 mg) on days −3, 0, 4, 8 from the start of tumor challenge. From day 8 on, anti-NK1.1 antibodies were injected once a week. All experiments were approved by the Animal Experiments Committee of the VUmc, Amsterdam.

### Tumor-infiltrating lymphocytes, spleens and tumor-draining lymph nodes

For isolation of tumor-infiltrating lymphocytes, tumors were removed from C57BL/6 mice, cut into grain size pieces and incubated in HE medium (RPMI medium with 10% FCS, 10 mM EDTA, 20 mM HEPES, 50 U/ml penicillin, 50 μg/ml streptomycin) supplemented with 1 WU/ml Liberase-TL (Roche Diagnostics GmbH, Manheim, Germany) and 50 μg/ml DNase I (Roche) for 30 min at 37°C. Erythrocytes were lysed with ACK lysis buffer and tumor-infiltrating lymphocytes were purified by ficoll gradient with Lymphoprep (Axis-Shield, UK).

Tumor-draining lymph nodes and spleens were passed through a 100 μm cell strainer (BD Falcon, NJ, USA) in Iscove's Modified Dulbecco's Medium (IMDM; Gibco, CA, USA) to generate single cell suspensions. Erythrocytes were lysed with ACK lysis buffer. Subsequently, the presence of different immune cells was analyzed upon staining with specific antibodies and flow cytometry. Alternatively, cells were re-stimulated with 30 μg/ml phorbol 12-myristate 13-acetate (PMA) and 500 ng/ml ionomycin (Sigma-Aldrich) for detection of IFN-γ in cell supernatants by ELISA (eBiosciences, CA, USA).

### Co-cultures of tumor cells and NK cells

To examine effects of sialylated tumorantigens on NK cell function, either total splenocytes or NK-enriched splenocytes derived from naive C57BL/6 mice were co-cultured at indicated E:T ratio with B16^SLC35A1^ or B16^scrambled^ tumor cells. Four hours after co-culture, the amount of IFN-γ in the supernatant was analysed by ELISA. To enrich for NK cells, splenocytes from naive mice were incubated with (rat-anti-mouse) antibodies directed against MHC-II, CD3, CD4, CD8, F4/80, MAC-1, followed by incubation with sheep-anti-rat antibody coated magnetic beads.

To determine effects of NK-derived IFN-γ on tumor-expressed chemokines and cytokines, B16^SLC35A1^ and B16^scrambled^ were cultured in the presence or absence of 250 pg/ml IFN-γ for 6 hours and expression of chemokines and cytokines was measured by qRT-PCR.

### Analysis of cell cycle, adhesion and motility

For cell cycle analysis by DNA content, tumor cells were collected and fixed in 70% ethanol o/n at 4°C. After washing, tumor cells were resuspended in PBS containing 10 μg/ml Propidium Iodide and 200 μg/ml RNase A and analyzed by flow cytometry.

For cell adhesion analysis, Nunc-immunoMaxisorp plates (Sigma-Aldrich) were coated o/n with 50 ulmatrigel (BD Biosciences) 1:25 in PBS buffer. After washing with TSM buffer (20 mMTris-HCl, pH 8, 150 mMNaCl, 1 mM CaCl_2_, 2 mM MgCl_2_), non-specific binding was blocked with TSM/0,5% BSA. CFSE-labeled tumor cells were allowed to adhere for 30 min at 37°C, and non-adherent cells were removed by gently washing plates three times with pre-warmed TSM/0.5% BSA. Subsequently, bound tumor cells were lysed using a 50 mMTris/0,1% SDS buffer and fluorescence was detected using a CytoFluor (Applied Biosystems, CA, USA). A motility or scratch assay was performed as previously described [38].

### Flow cytometry

The following monoclonal antibodies (derived either from BD biosciences or eBioscience) were used: FITC-labeled anti-CD3 (145-2C11), -CD4 (RM4-5), -NK1.1 (PK136 ebio), -MHC-I (H-2Kb; AF688.5BD),-CD8b (eBIOH35); PE-labeled anti-CD8b (eBioH35-17.2), -MHC-II (M5/114.15.2) and −NK1.1 (PK136); and APC-labeled anti-FoxP3 (FJK-169). Biotin-labeled plant-derived lectin *SambucusNigra (SNA*) was obtained from Vector Labs, CA, USA, and binding was detected with PE-labeled Streptavidin (Jackson Immunoresearch, UK).

### qRT-PCR

Total RNA was extracted using the RNeasy kit (Qiagen, Valencia, CA) combined with a DNase treatment to remove contaminating DNA (Qiagen). cDNA was synthesized using the Reverse Transcription System kit (Promega, WI, USA) following manufacturer's guidelines. Real time PCR reactions were performed using the SYBR Green method in an ABI 7900HT sequence detection system (Applied Biosystems), with GAPDH as internal control. Samples were analyzed in triplicate and normalized to GAPDH. Primers were obtained from Invitrogen (Carlsbad, California) and sequences were as follows: GAPDH-FWD:GACAACTCATCAAGATTGTCAGCA;GAPDH-REV:TTCATGAG CCCTTCCACAATG;IL-10-FWD:GGACAACATACTG CTAACCG;IL-10-REV:GGGGCATCACTTCTACCAG; TGFβ-1-FWD:GCTGAACCAAGGAGACGGAATA; TGFβ-1-REV:GGGCTGATCCCGTTGATTT;IP-10-FWD:GACGGTCCGCTGAACTG;IP-10-REV:GCTTCC CTATATGGCCTCATT;IFNg-R-FWD:GCTTTGACGAG CACTGAGGA;IFNg-R-REV:CCAGCATACGACAGGG TTCA;SLC35A1-FWD:CATGGCCTTCCTGGCTCTC; SLC35A1-REV:GGTCACCTGGTACACTGCTGC.

### Statistical analysis

Prism software (GraphPad 5.0) was used for statistical analysis. Student's *t* test was to determine statistical significance. Statistical significance for all the tests, assessed by calculating the *P* values, was defined as *P* < 0.05.

## SUPPLEMENTARY MATERIALS FIGURE


