# Supplementary material for: Tumor sialylation impedes T cell mediated anti-tumor responses while promoting tumor associated-regulatory T cells
Source: Oncotarget. 2016 Jan 5;7(8):8771–82. doi: 10.18632/oncotarget.6822 (PMC4891003; doi:10.18632/oncotarget.6822)
Supplement: Supplementary file 1 [file oncotarget-07-8771-s001.pdf]

Tumor sialylation impedes T cell mediated anti-tumor responses while promoting tumor associated-regulatory T cells

Supplementary Materials

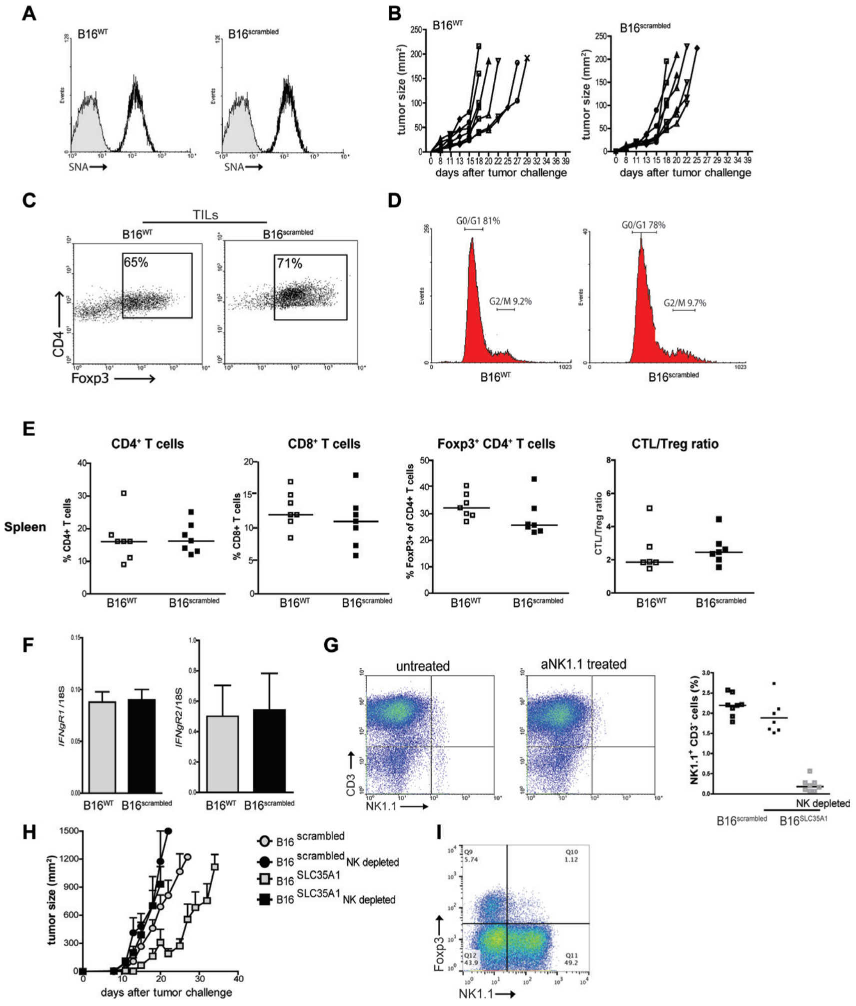

Supplementary Figure S1: B16^{scrambled} behaves similar to WT B16 *in-vitro* and *in-vivo*. (**A**) Detection of α2,6-sialic acids using SNA on B16^{wt}, B16^{scrambled} and B16^{SLC35A1} tumors (black lines) by flow cytometry. Gray filled histograms represent conjugate control. Analysis of sialylation was performed at least three times. (**B**) Tumor growth in B16^{wt} and B16^{scrambled}tumor-bearing mice (*n* = 7 mice/group). Tumor growth assessed at different days after tumor inoculation is indicated as mm^2 of tumor volume (mean ± s.e.m). (**C**) Analysis of intra-tumoral Foxp3$^+$CD4$^+$ T cells in B16^{wt} and B16^{scrambled} tumors by flow cytometry. Percentage of Foxp3$^+$ of CD4$^+$ T cells in a representative mouse of each group is shown. (**D**) Cell cycle analysis of B16^{wt} and B16^{scrambled} tumors by DNA content. Percentage of cells in G0/G1 interphase and G2/M mitotic phase are indicated. Results are representative of 3 experiments. (**E**) Spleens of mice challenged with eitherB16^{SLC35A1} or B16^{scrambled} tumor cells were analyzed by flow cytometry to determine the frequency of CD4$^+$ and CD8$^+$ T cells and of Foxp3$^+$CD4$^+$ T cells. CTL/Treg ratios were calculated for each mouse. Dots represent individual mice (*n* = 7). Bars indicate median of each group. Plots represent 2 experiments. (**F**) Expression of IFN-y receptor (IFNGR)in B16^{SLC35A1} and B16^{scrambled} tumors as determined by qRT-qPCR. Gene expression was normalized and presented as relative expression to GAPDH. Values shown are the mean ± s.e.m of two experiments. (**G**) Detection of NK cells, distinguished as NK1.1$^+$ CD3$^-$ cells, in spleens from untreated and anti-NK1.1-treated tumor-bearing mice (*n* = 7/8 per group) by flow cytometry; representative plots are shown (left). The percentage of NK1.1$^+$ CD3$^-$ cells (median) is represented in a graph (right). (**H**) WT and NK-depleted mice were challenged with B16^{SLC35A1} or B16^{scrambled} tumor cells. Tumor growth, assessed every 2–3 days after tumor inoculation, is indicated as mean mm^2 ± s.e.m; *n* = 7−9 mice/group. (**I**) Analysis of NK1.1 expression by Foxp3$^+$ T cells in TILs of B16^{scrambled} tumors (*n* = 6 mice). CD45$^+$ cells were gated and expression of NK1.1 and Foxp3 is shown. A representative example is shown.
